# Mechanism of Action of Formate Dehydrogenases

**DOI:** 10.1021/jacs.4c07376

**Published:** 2024-10-09

**Authors:** Dimitri Niks, Sheron Hakopian, Alexa Canchola, Ying-Hsuan Lin, Russ Hille

**Affiliations:** † Department of Biochemistry, University of California, Riverside, California 92521, United States; ‡ Department of Environmental Sciences, University of California, Riverside, California 92521, United States

## Abstract

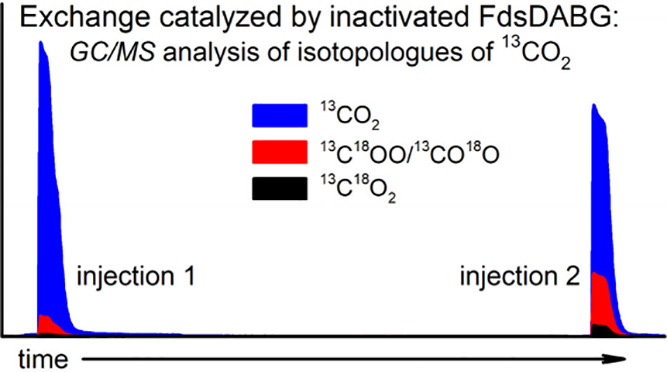

The molybdenum- and tungsten-containing formate dehydrogenases from a variety of microorganisms catalyze the reversible interconversion of formate and CO_2_; several, in fact, function as CO_2_ reductases in the reverse direction under physiological conditions. CO_2_ reduction catalyzed by these enzymes occurs under mild temperature and pressure rather than the elevated conditions required for current industrial processes. Given the contemporary importance of remediation of atmospheric CO_2_ to address global warming, there has been considerable interest in the application of these enzymes in bioreactors. Equally important, understanding the detailed means by which these biological catalysts convert CO_2_ to formate, a useful and easily transported feedstock chemical, might also inspire the development of a new generation of highly efficient, biomimetic synthetic catalysts. Here we have examined the ability of the FdsDABG formate dehydrogenase from *Cupriavidus necator* to catalyze the exchange of solvent oxygen into product CO_2_ during the course of formate oxidation under single-turnover conditions. Negligible incorporation of ^18^O is observed when the experiment is performed in H_2_^18^O, indicating that bicarbonate cannot be the immediate product of the enzyme-catalyzed reaction, as previously concluded. These results, in conjunction with the observation that the reductive half-reaction exhibits mildly acid-catalyzed rather than base-catalyzed chemistry, are consistent with a reaction mechanism involving direct hydride transfer from formate to the enzyme’s molybdenum center, directly yielding CO_2_. Our results are inconsistent with any mechanism in which the initial product formed on oxidation of formate is bicarbonate.

It is known that both the molybdenum-containing *E. coli* FdhF formate dehydrogenase (a component of the organism’s fully reversible formate:hydrogen lyase complex)^5^ and the molybdenum-containing *Cupriavidus necator* FdsDABG formate dehydrogenase^6^ catalyze the transfer of the C_α_ hydrogen of substrate to their metal centers in the course of the reaction, reducing their L_2_Mo^VI^S(SeCys) and L_2_Mo^VI^S(Cys) active sites to L_2_Mo^IV^(SH)(SeCys) and L_2_Mo^IV^(SH)(Cys), respectively (here L represents a pyranopterin cofactor coordinated to the metal in a bidentate fashion by an enedithiolate side chain).^1−4^ On the basis of these observations, a reaction mechanism has been proposed involving a simple hydride transfer from substrate concomitant with formation of a second C=O bond to make CO_2_,^6^ as shown in Figure 1. Such a mechanism readily accounts for the observed reversibility of the reaction. A key element of the mechanism is that the metal center is coordinationally stable, with no dissociation of the (Se)Cys ligand provided by the polypeptide. This mechanism has been widely accepted in the field and is consistent with other work that has concluded that CO_2_ is the direct product of the fully reversible reaction in this and related systems.^5,7−10^ In the case of the *C. necator* FdsDABG formate dehydrogenase, the reaction has been shown to be fully reversible, with the steady-state *K*_m_ and *V*_max_ values in the forward and reverse directions complying with the expected Haldane relationship.^11,12^ It is likely that all formate dehydrogenases (as well as the closely related formylmethanofuran dehydrogenases) are fully reversible when care is taken to use CO_2_ rather than bicarbonate as substrate.^1,12^ Recent work, however, with the *Rhodobacter capsulatus* FdsDABG, closely related to the *C. necator* enzyme, has concluded that the initial product of the reaction is bicarbonate rather than CO_2_, and an alternate mechanism has been proposed (Figure 1) in which the enzyme first hydroxylates formate to bicarbonate in a manner similar to the reaction catalyzed by xanthine oxidase, followed by dehydration to CO_2_.^13^

**Figure 1:**
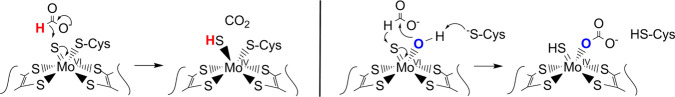
Proposed mechanisms for the reaction catalyzed by formate dehydrogenases. *Left*, that proposed by Niks et al.,^6^ involving a simple hydride transfer from formate to the active site molybdenum center, resulting in direct formation of CO_2_. *Right*, that proposed by Kumar et al.,^13^ involving displacement of cysteine from the molybdenum coordination sphere by solvent-derived hydroxide, followed by base-assisted nucleophilic attack on the substrate carbonyl to yield bicarbonate coordinated to the molybdenum via the catalytically introduced hydroxyl group. The bicarbonate subsequently dehydrates to CO_2_ prior to exiting the active site, regenerating the water in the active site.

The reaction is proposed to proceed by solvent hydroxide displacing cysteine from the molybdenum coordination sphere, the dissociated cysteinate then serving to deprotonate the Mo-OH to initiate nucleophilic attack on the substrate carbonyl to yield bicarbonate coordinated to the metal by the catalytically introduced hydroxyl. A key piece of evidence in support of this alternate reaction was the reported incorporation of solvent ^18^O into product CO_2_ when the reaction was carried out in H_2_^18^O, as examined by gas chromatography/mass spectrometry (GC/MS). In fact, less than 20% of that expected given the level of ^18^O enrichment in solvent was observed in the course of catalysis, with the observation being explained by invoking an active site water molecule that was unable to exchange with solvent. Under multiple turnover conditions, it was argued that the initially incorporated ^18^O would be quickly washed out and would therefore not show up in product. (Upon dehydration of the bicarbonate generated in the course of the reaction according to this mechanism, there is a two in three chance that one of the two ^16^O atoms of product will be lost, regenerating the hypothesized water trapped in the active site, which will then be unlabeled; subsequent turnovers would involve trapped water progressively de-enriched in ^18^O, resulting in no ^18^O appearing in product.)

There are several issues with this alternate mechanism (among them that the proposed catalytic water is seen in only some of the crystal structures of various formate dehydrogenases and there being no structural support for the displacement of cysteine from the molybdenum coordination sphere by hydroxide in the course of catalysis), but given the importance in understanding the specific manner by which formate dehydrogenases catalyze the facile interconversion of formate and CO_2_, we have reexamined whether the enzyme is able to catalyze the incorporation of solvent oxygen into product CO_2_ by mass spectrometry. There are three critical differences between the previous work and that described here: (1) we have utilized single-turnover conditions so that, in the event there is a water molecule trapped in the active site that exchanges only slowly, any label incorporated into product CO_2_ on oxidation of formate should be detected at or near the theoretical level; (2) we have used 70% enriched H_2_^18^O rather than the 10% H_2_^18^O used previously to make it easier to detect any ^18^O incorporated into product; and (3) our experiments have been performed at pH 7.5 rather than 9.0 to favor the dehydration of any catalytically generated bicarbonate, since it is the vapor phase that is being experimentally analyzed.

To permit distinction between enzymatically generated CO_2_ due to oxidation of formate from atmospheric CO_2_, ^13^C-labeled formate was used as a substrate. Concentrated enzyme (typically 630 μM) was reacted on ice in a sealed vessel at pH 7.5 with one equivalent of sodium formate that was doubly labeled with ^13^C (99% enrichment) and ^2^H (98%), the latter substitution used so as to slow the enzyme-catalyzed reaction; the ^18^O enrichment in solvent water was 70%. The headspace of the vessel was analyzed for ^13^C^16^O_2_, ^13^C^18^O^16^O/^13^C^16^O^18^O, and ^13^C^18^O_2_ (with *m*/*z* 45, 47 and 49, respectively), withdrawing samples at 30 s (the shortest time possible) and 70 s after initiating reaction by addition of substrate. At 30 s only 16% exchange is evident, and by 70 s that exchange increases to ∼39% (Figure 2, *left*). Had the enzyme catalyzed ^18^O incorporation into product CO_2_ from solvent, the expectation would be that bicarbonate formed enzymatically would result in 47% exchange at the completion of the single turnover. Our results indicate that, while there was a significant amount of noncatalytic exchange of ^18^O into CO_2_ under our experimental conditions, this exchange occurred almost entirely subsequent to the generation of the enzyme-catalyzed CO_2_, which under the present experimental conditions is expected to be complete within 200 ms. This slow, postcatalytic exchange has been observed previously^13^ but is considerably faster than extrapolating back to such short times—essentially all the exchange seen at 30 s is due to the postcatalytic exchange that continues to 70 s. Importantly, this was considerably faster than the known exchange rate of ^18^O into CO_2_, which occurs with a half-time >5 min at pH 7.5 on ice (estimated from Johnson,^14^ their Figure 3 describing the dependence of exchange rate on temperature; and Ho and Sturtevant,^15^ their Figure 2 presenting the exchange rate as a function of pH), meaning that any label introduced by enzyme would not exchange back out in the time frame of our experiments. In control experiments, we found no noncatalytic exchange of ^18^O from solvent into ^13^CO_2_ under the present experimental conditions in the first 30 s of reaction above instrument background and only <1% additional exchange after 70 s of reaction (Figure 2, *left*, controls). Finally, to be certain that the mass spectrometer was not biased to one or another isotopologue of CO_2_ (*m*/*z* 45, 47, or 49), standard curves were constructed with sodium bicarbonate-derived ^13^CO_2_ equilibrated with 5% H_2_^18^O (Figure 2, *right*).^16^

**Figure 2:**
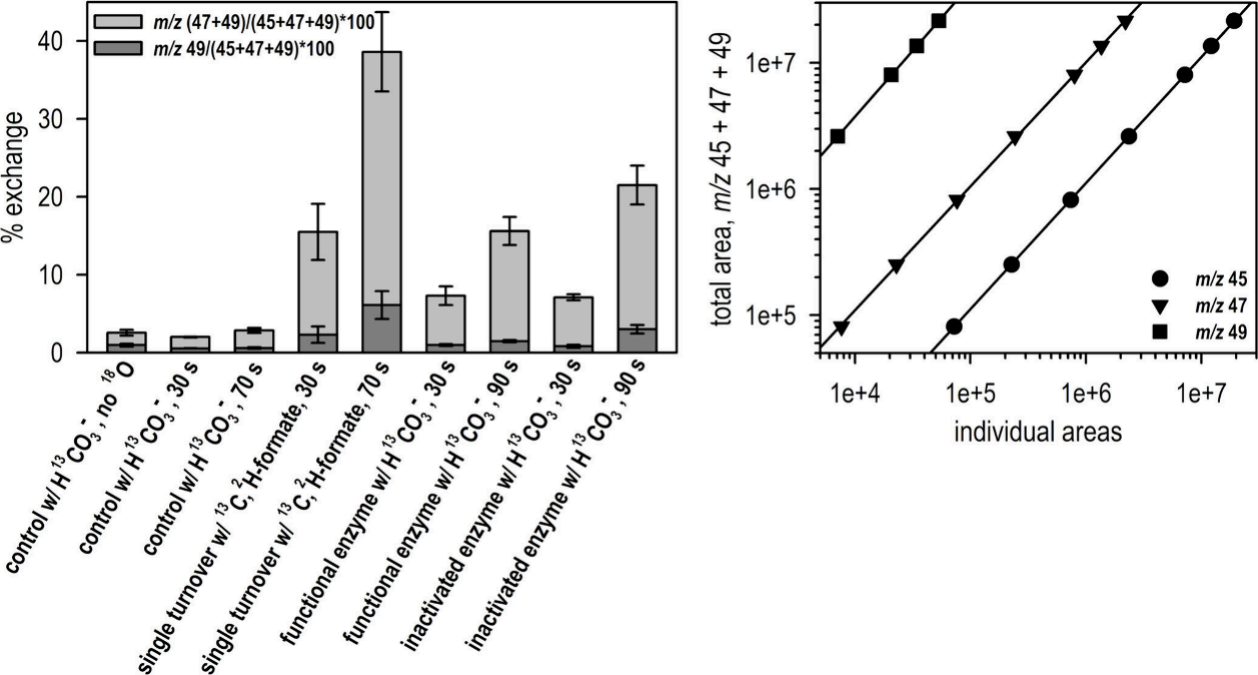
^18^O exchange, as quantified by GC/MS. *Left*, Summary of the results of the exchange experiments under various conditions. All of the experiments were performed on ice in 20 mM HEPES, pH 7.5, containing 0.1% Antifoam B and 70% H_2_^18^O (except for the control sample which did not contain any H_2_^18^O). All experiments were performed at least in triplicate. *Right*, Standard curves^16^ plotted on a double logarithmic axis for the sodium bicarbonate-derived ^13^CO_2_ equilibrated overnight with 5% H_2_^18^O. The slope of the regression was 0.999, 0.983, and 1.04 for *m*/*z* 45, 47 and 49, respectively, and the calculated exchange for the *m*/*z* 45 and 47 isotopologues was 10.1 ± 0.39% (*n* = 4), in good agreement with the expected exchange of 10%. (To arrive at the expected percentage, the ^18^O content is adjusted by the ×2 combinatorial multiplier for a two-site exchange: 5% for each exchangeable site, as given by eq 1 in ref (18). The same expected ratio is obtained by taking into account the fact that exchange occurs at three equivalent oxygens in bicarbonate: here the ^18^O content is adjusted by the ×3 combinatorial multiplier for a three-site exchange to give 15%, further modified by 2/3 to account for the likelihood that the newly labeled oxygen is lost upon dehydration, yielding the expected 10%.)

To ascertain whether the observed postcatalytic ^18^O exchange from solvent seen here required functional protein, concentrated FdsDABG (either active or inactivated by reaction with potassium cyanide) was reacted with a stoichiometric concentration of ^13^C-labeled bicarbonate to mimic the single-turnover conditions. With the functional protein, exchange occurred at a lower level than in the single-turnover experiment, increasing from 6% at 30 s to 14% at 90 s. The reduced extent of exchange compared to the single-turnover condition is consistent with the low availability of CO_2(aq)_ at pH 7.5, where the equilibrium heavily favors bicarbonate,^17^ and is not due to exchange being limited by access of CO_2(aq)_ to a hypothetical site of exchange. This is evident by the significant amount of the doubly labeled ^13^C^18^O_2_ with *m*/*z* 49, present in some of the experiments (Figure 2, *left*). Surprisingly, the exchange persisted when inactivated enzyme was used, and at approximately the same level as in the homologous experiment with the functional enzyme. These results indicate that the postcatalytic exchange is clearly a side reaction and not a feature of the catalytic conversion of formate to CO_2_.

Another difference between the two mechanisms shown in Figure 1 is that a direct hydride transfer mechanism does not involve acid–base catalysis, whereas the bicarbonate mechanism does. We therefore examined the pH dependence of the reductive half-reaction of FdsDABG, i.e., its reduction by formate. We have previously demonstrated that the steady-state *k*_cat_ exhibits a bell-shaped pH dependence, with apparent p*K*_a_’s for the ascending and descending limbs of approximately 6.0 and 9.0, respectively.^6^ Given the likelihood that the rate-limiting step in overall catalysis does not reside in the reductive half-reaction, however, it is necessary to examine the kinetics of the reduction of enzyme by formate using rapid reaction kinetics. At pH 7.7, the overall reaction of FdsDABG with a pseudo-first-order excess of formate is multiphasic, as expected since the enzyme possesses seven iron–sulfur clusters and an FMN in addition to its molybdenum center, meaning that six equivalents of formate are required for the reaction to go to completion.^6^ The fastest phase of the reaction exhibits hyperbolic dependence on [formate], as expected for a reaction in which the substrate must reversibly bind to the enzyme prior to its oxidation, with a *k*_fast_ of 140 s^–1^ and *K*_d_^formate^ of 82 μM at 10 °C. Figure 3 shows a plot of the extrapolated *k*_fast_ at high [formate] over the pH range from 6 to 9. It is abundantly clear that the reaction exhibits only mild acid catalysis (decreasing from ∼400 s^–1^ to ∼200 s^–1^ with a p*K*_a_ ≈ 7) rather than strong base catalysis.

**Figure 3:**
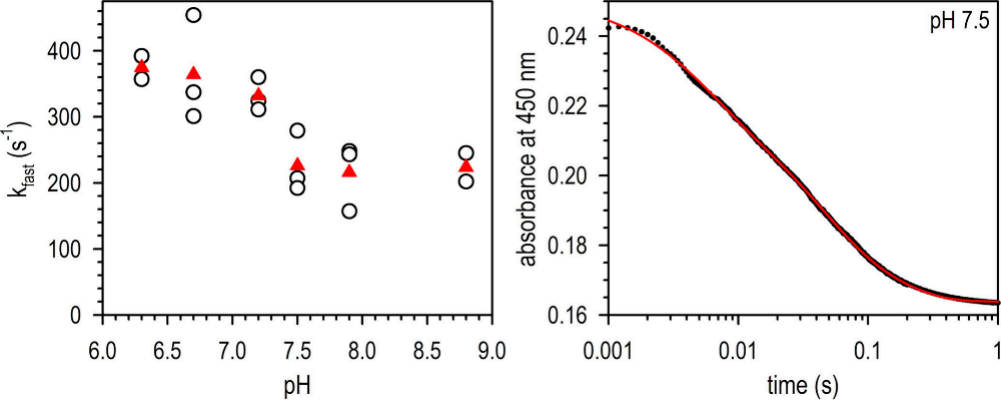
pH dependence of the fastest phase of the reduction of FdsDABG by excess formate. *Left*, Plotted are the limiting *k*_fast_ values at high [formate] as a function of pH (black circles) and the average of individual rate constants for each pH (red triangles). The pH profile reflects acid catalysis in the reductive half-reaction, with a p*K*_a_ of ∼7. *Right*, A representative kinetic trace of the reaction at pH = 7.5 (black dots) and the corresponding fit (red line). The trace is best represented by three phases. Details of the fitting procedure are described in the Supporting Information.

On the basis of the above, we conclude that FdsDABG oxidizes formate in a way that in fact does not involve incorporation of oxygen from solvent into product CO_2_, eliminating the possibility that bicarbonate rather than CO_2_ is the initial product formed in the reaction and indicating that there is no catalytically important water molecule trapped in the enzyme’s active site. This being the case, other results invoked in support of a bicarbonate mechanism by these workers require reinterpretation. On the other hand, our results are fully consistent with a direct hydride transfer mechanism (Figure 1, *left*).

## Supporting Information Available

Experimental Section describing reagents and methods for protein expression/purification, rapid reaction kinetics, and GC/MS (PDF)
