# Supplementary material for: Mechanism of Action of Formate Dehydrogenases
Source: J Am Chem Soc. 2024 Oct 9;146(42):28601–4. doi: 10.1021/jacs.4c07376 (PMC11503769; doi:10.1021/jacs.4c07376)
Supplement: Supplementary file 1 — ja4c07376_si_001.pdf [file ja4c07376_si_001.pdf]

On the Mechanism of Action of Formate Dehydrogenases

Dimitri Niks[a], Sheron Hakopian[a], Alexa Canchola[b], Ying-Hsuan Lin[b] and Russ Hille[a*]

[a] Department of Biochemistry, University of California, Riverside, CA 92521

[b] Department of Environmental Sciences, University of California, Riverside, CA 92521

* To whom correspondence should be addressed: russ.hille@ucr.edu

Keywords: Formate dehydrogenase, Hydride transfer, Oxygen exchange

SUPPORTING INFORMATION

EXPERIMENTAL SECTION

Reagents. UHP helium (99.999%, Airgas) was used as inert carrier gas in the GC/MS work. 99% ^{13}C sodium bicarbonate (CLM-441-1) and ^{13}C (99%), ^{2}H (98%) sodium formate (CDLM-6203-0.25) were purchased from Cambridge Isotope Labs; >98% $H_2{}^{18}O$ was purchased from Olinax Inc. Antifoam B emulsion (A5757) and catalase (C-40) were purchased from Sigma-Aldrich and superoxide dismutase (LS003541) was purchased from Worthington Biochemical Corp. GC vials (29432-U), inserts (29441-U) and caps with PTFE/silicone septa (27273) were purchased from Supelco.

FdsDABG expression. DH5α *E. coli* cells containing the pTrc-strep-FdsGBACD plasmid were grown as previously described[1] with minor modifications. A pre-culture in Terrific Broth (TB) medium supplemented with 100 μg/mL ampicillin and 1 mM sodium molybdate was grown overnight at 37°C. The next morning, this pre-culture was used to inoculate 6 L Erlenmeyer flasks, each containing 3 L of TB medium supplemented with 100 μg/mL ampicillin, 1 mM sodium molybdate, 24 mg ferric ammonium citrate, and 45 μM isopropyl-β-D-1-thiogalactopyranoside (IPTG) to starting $OD_{600\ nm}$ = 0.002. The flasks were grown at 25°C and 160 rpm in a refrigerated incubator shaker (Innova 4335, New Brunswick) to a final $OD_{600\ nm}$ = 6 - 7 (~ 30 hrs). Harvested cells were frozen in liquid nitrogen and stored at -80°C.

Purification of FdsABG. Purification of FdsDABG was performed as previously described[1] except that the following modified gradient elution procedure was used to separate the functional FdsDABG from non-functional protein and from FdsBG: elution from the Strep column was applied to a 2.6 x 8 cm Fractogel TMAE 650 (S) column (EMD Millipore), washed with 0.5 column volumes of 50 mM potassium phosphate, 10 mM KNO_3, pH 7.0, and eluted with a linear gradient of 16% - 20.5% 1 M NaCl-containing 50 mM potassium phosphate, 10 mM KNO_3, pH 7.0 over 7 column volumes. Enzyme thus purified exhibited >70 units of activity per mg of protein. Purified protein was stored in liquid nitrogen in 75 mM K-PO_4, 10 mM KNO_3, pH 7.5 (long term) or at -80◦C as a 50% ammonium sulfate precipitate (short term).

Rapid Reaction Kinetics. The effect of pH on the reaction of FdsDABG with formate was followed using an Applied Photophysics, Inc. SX-20 stopped-flow spectrophotometer equipped with a photomultiplier (PMT) detector and running ProData SX 2.2.27 acquisition software. An overlapping buffer system similar to that previously described[2] consisting of 75 mM each of maleate, K_2HPO_4, Tris, and glycine to cover pH range of ~6.0 - 9 was employed. Final pH was verified by diluting buffer 5-fold. An ammonium sulfate precipitate of >90% active FdsDABG was resuspended in 20 mM HEPES, 10 mM KNO_3, pH 7.5 in the anaerobic chamber (Coy Laboratory Products). A PD-10 desalting column (Cytiva) was used to remove KNO_3 and the enzyme was diluted into buffer with corresponding pH prior to being transferred to the stopped-flow spectrophotometer in a tonometer. 4 μM FdsDABG was then reacted at 10°C with 4 mM sodium formate (a saturating substrate concentration over the experimental pH range). Time courses for the reaction were monitored at 450 nm fitted to a sum of three exponentials by nonlinear least squares regression analysis to the following equation:

$$A_t = A_\infty \pm \sum A_n \exp(-t/k_n) \tag{1}$$

where n refers to the number of kinetic phases observed. Analysis of time courses was performed using the software ProData Viewer 4.2.0 (Applied Photophysics, Inc). Only the fastest observed rate constants, k_{fast}, corresponding to the initial rate of reduction were plotted against pH.

GC/MS. GC/MS analysis of CO_2 was performed using an Agilent 6890N gas chromatograph coupled to an Agilent 5975C single quadrupole mass spectrometer. To minimize the effect of background exchange in the instrument, the traditional capillary column was replaced with deactivated tubing (Restek Hydroguard, 5 m x 0.18 mm i.d.). The following GC conditions were used: 1:10 split injection with 120°C injector temperature, 30°C oven temperature with no temperature ramping, and 2 ml/min flow rate with. helium as carrier gas. Data were acquired in selected ion monitoring (SIM) mode (*m/z* 45, 47, and 49) at high resolution (+/- 0.3 *m/z* units). Peak integration was performed using OpenChrom 1.5.0 software (Lablicate). To insure that the mass spectrometer was not biased to one or another isotopologue of CO_2 (*m/z* 45, 47, or 49), standard curves were constructed by injecting various amounts of $^{13}CO_2$ derived from a solution of ^{13}C bicarbonate (equilibrated in 5% $H_2{}^{18}O$ overnight)[3].

All experiments were performed on ice in 20 mM HEPES, pH 7.5 containing 0.1% Antifoam B and 70% $H_2{}^{18}O$ (except for the control sample which did not contain any $H_2{}^{18}O$). The addition of antifoam to all of the samples insured that no liquid ended up in the gastight syringe during headspace withdrawal described below. All protein samples and substrates were made anaerobic prior to reaction on an anaerobic train with ALPHAGAZ grade Ar (Airgas, 10 ppb O_2). To obtain sufficient $^{13}CO_2$ for accurate MS detection (extraction of CO_2 gas from samples on ice at pH 7.5 is particularly challenging since most of the CO_2 in solution is in the form of bicarbonate[4]) samples sealed in GC vials with 0.5 ml inserts and caps with PTFE/silicone septa were placed under a slight vacuum by removing 1.5 ml of headspace with a 2.5 ml SampleLock gastight syringe (Hamilton) prior to injection of substrate. After mixing the protein with substrate (either ^{13}C, ^{2}H sodium formate or ^{13}C-labeled bicarbonate) an additional 2.5 ml of headspace was withdrawn from the GC vial. The syringe was then locked to prevent gas escape, removed from the

reaction vessel, the plunger compressed to ~0.2 ml and then the gas injected into the GC (30 s injection). To monitor the progression of the exchange an additional 2.5 ml of headspace was withdrawn, compressed and injected at 70-90 s.

FdsDABG was first buffer-exchanged into anaerobic 20 mM HEPES, pH 7.5, in the presence of 100 units / ml superoxide dismutase and 1.5 nM catalase via 50 kDa MWCO Amicon Ultra - 4 (EMD Millipore). It has been previously shown that FdsDABG is resistant to inactivation by oxygen in the presence of superoxide dismutase even in the absence of the inhibitor KNO_3[1]. In this way protein can be prepared semi-aerobically with <15% loss in activity. After removal of KNO_3, FdsDABG was diluted to 70% $H_2{}^{18}O$, made anaerobic and transferred to the anaerobic chamber. There it was supplemented with 0.1 % Antifoam B, aliquoted and sealed in capped GC vials with inserts. The overall procedure took >3 hrs, allowing $H_2{}^{18}O$ sufficient time to exchange into the active site of the enzyme (incubation over a 3-day period did not yield results that were significantly different; data not shown). For single-turnover experiments 45 μl of 0.6 mM ^{13}C, ^{2}H sodium formate in 70% $H_2{}^{18}O$ was injected into GC vials containing 45 μl of 0.63 mM functional protein. To mimic conditions for the single-turnover conditions, experiments with ^{13}C-labeled bicarbonate were performed at approximately 1:1 ratio of CO_2 to protein. To that end 2 μl of 50 mM ^{13}C-labeled bicarbonate in anaerobic water was injected into 100 μl of 0.59 mM enzyme (either functional or inactivated) and quickly mixed. Non-functional enzyme was prepared by incubation for 1 hr with 2 mM potassium cyanide[5] (terminal sulfur ligands to the Mo cofactor are known to be susceptible to cyanolysis). Potassium cyanide was removed via Amicon Ultra - 4 and the protein once again diluted to 70% $H_2{}^{18}O$ in preparation for the experiment with ^{13}C-labeled bicarbonate described above.

REFERENCES

[1] Hakopian, S., Niks, D., and Hille, R. (2022) The air-inactivation of formate dehydrogenase FdsDABG from *Cupriavidus necator*., *Journal of Inorganic Biochemistry 231*:111788. https://doi.org/10.1016/j.jinorgbio.2022.111788

[2] Niks, D., Duvvuru, J., Escalona, M., and Hille, R. (2016) Spectroscopic and Kinetic Properties of the Molybdenum-containing, NAD(+) - dependent Formate Dehydrogenase from Ralstonia eutropha, *Journal of Biological Chemistry 291*, 1162-1174.

[3] Isobe, K., Koba, K., Ueda, S., Senoo, K., Harayama, S., and Suwa, Y. (2011) A simple and rapid GC/MS method for the simultaneous determination of gaseous metabolites, *Journal of Microbiological Methods 84*, 46-51.

[4] Butler, J. N. (1982) *Carbon Dioxide Equilibria and Their Applications.*, Addison-Wesley, Reading, MA. p. 23.

[5] Massey, V., and Edmondson, D. (1970) The mechanism of inactivation of xanthine oxidase by cyanide., *Journal of Biological Chemistry 245*, 6595-6598.
